# Improvement in Fatigue Performance of Aluminium Alloy Welded Joints by Laser Shock Peening in a Dynamic Strain Aging Temperature Regime

**DOI:** 10.3390/ma9100799

**Published:** 2016-09-26

**Authors:** Chun Su, Jianzhong Zhou, Xiankai Meng, Shu Huang

**Affiliations:** 1 School of Mechnical Engineering, Jiangsu University, Zhenjiang 212013, China; zhoujz@ujs.edu.cn (J.Z.); xiankaimeng@gmail.com (X.M.); huangs@ujs.edu.cn (S.H.); 2 School of Mechanical & Vehicle Engineering, Changzhou Institute of Technology, Changzhou 213000, China

**Keywords:** laser shock peening, AA6061-T6 welded joint, dynamic strain aging, residual stress, high-cycle fatigue, fracture

## Abstract

As a new treatment process after welding, the process parameters of laser shock peening (LSP) in dynamic strain aging (DSA) temperature regimes can be precisely controlled, and the process is a non-contact one. The effects of LSP at elevated temperatures on the distribution of the surface residual stress of AA6061-T6 welded joints were investigated by using X-ray diffraction technology with the sin^2^*ϕ* method and Abaqus software. The fatigue life of the welded joints was estimated by performing tensile fatigue tests. The microstructural evolution in surface and fatigue fractures of the welded joints was presented by means of surface integrity and fracture surface testing. In the DSA temperature regime of AA6061-T6 welded joints, the residual compressive stress was distributed more stably than that of LSP at room temperature. The thermal corrosion resistance and fatigue properties of the welded joints were also improved. The experimental results and numerical analysis were in mutual agreement.

## 1. Introduction

Aluminum (Al) alloys, as light-weight, high-strength, corrosion-resistant metals, have always been applied to the manufacture of aircraft. In recent years, with the increasingly higher requirements imposed upon light-weighting of automobiles, Al alloys have begun to be used to fabricate parts of automobiles including chassis and bumpers. At present, they have been used in industrial production instead of merely in laboratory research. For instance, BMW i3 cars with a chassis made of Al alloy are produced in large quantities and ultimately reach half of the conventional capacity of counterpart products [1]. Welding aluminum alloys using friction stir welding (FSW) has brought significant benefits [2,3]; however, it has disadvantages such as being only applicable to works involving rigid fixation and rapid loss of a stirrer. Hence, traditional techniques including tungsten inert gas (TIG) welding are also widely used in actual production. AA6061-T6 is an Al alloy with good weldability. However, joints welded with this kind of alloy using conventional welding methods, including tungsten inert gas (TIG) welding, have a strength far lower than that of the base metal, thus significantly affecting the service life of weldments [4]. This is because intergranular cracks tend to be formed in the material owing to the presence of welding heat-affected zones (HAZs). Meanwhile, defects, such as air holes and impurities, in the weld bead can easily lead to stress concentrations and therefore generate crack sources [5]. As a consequence, the microstructure and stress state of weld beads and HAZs are changed, thereby effectively improving the fatigue performance of Al alloy weldments.

As laser technology has developed rapidly in recent years, laser shock peening (LSP) is now applied in the post-processing enhancement of metal weld joints. NASA’s Johnson Space Center in the USA verified that LSP can substantially reduce stress corrosion and the crack growth rate of weld beads. In addition, this technique has been used to reinforce the weld joints of nuclear waste containers [6,7]. Zhang et al. strengthened welded joints made of AISI304 steel using LSP, and enhanced the yield strength and fatigue life by 121.79% and 102.87%, separately [8]. Based on experimental results, L. Zhang validated the strengthening effect of double shot peening on welded joints of AISI304 steel as superior to that using single shot peening [9]. S. Huang et al. studied the influence of LSP with different laser energies in different peening areas on the surface residual stress and crack growth rate of an AA6061-T6 Al alloy [10,11]. During the past few years, strengthening effects have also been obtained in the LSP of AA6061-T6 welded joints [12].

In the above investigations, plastic deformation and dense dislocation happen on the surface of the metal workpieces under the effect of laser shock waves. In this way, the structural strength of and stresses on the workpieces are increased, thus improving their fatigue life [13,14]. However, the residual compressive stress fields induced by LSP are unstable under alternating loads, and therefore the stress can easily be relieved [15,16]. Existing studies show that, within a certain range of strain rates and temperatures, metal materials suffer from dynamic strain aging (DSA). This means that high density dislocation and dislocation pinning happen in metals and alloys due to the interaction between the moving solute atoms and dislocations. Meanwhile, repeated crystallization occurs, giving rise to partial cellular dislocation under the effect of strain, thus forming grains or sub-grains. Consequently, ultra-fine-grained structures are formed. The strengthening effects, including high-density dislocation, dislocation pinning, and grain refinement, can improve the cycling stability and thermostability of the microstructures of materials. Moreover, they are able to reduce the relaxation of residual compressive stress under cyclic loads at high temperatures [17,18]. LSP, assisted by DSA, combines LSP with thermomechanical coupling effects. It can generate high-density dislocation and residual compressive stress with a high amplitude. Besides, the high stability of residual stress and surface strength under elevated temperatures and cyclic loading ensures the reliability of the improvement in fatigue performance [19,20]. In recent years, attention has been paid to the research of this new technology. C. Ye et al. performed LSP on a 6061 Al alloy at a heating temperature of 160 °C and studied the microstructure and high-density dislocation behavior of the material. The acquired results indicate that due to the dislocation pinning effect induced by the precipitation of nanoparticles, the release rate of residual compressive stress generated by LSP at high temperatures is decreased [21]. An analysis of the strengthening effect on AISI4140 steel using LSP at 250 °C reveals that the combined effect of DSA and dynamic precipitation in the LSP assisted by temperature stabilizes the dislocation structure through pinning of dislocation slip defects. Thus, the fatigue performance and thermostability of this material are superior to those found with LSP at room temperature [22]. Gujba et al. pointed out that LSP assisted by DSA can overcome the effects of stress relaxation which is easily generated in LSP at room temperature. Nevertheless, good strengthening effects can only be obtained in the LSP assisted by DSA when selecting the correct working temperature and constraining media [23]. At present, no studies have been reported on the strengthening of Al alloy weldments using LSP assisted by DSA. Therefore, it is necessary to study the effect of LSP assisted by DSA on the fatigue performance of Al alloy welded joints.

This research performed LSP on the weld beads and HAZs of AA6061-T6 welded joints at DSA temperature. The tensile fatigue performances of these joints were detected before, and after, LSP, as assisted by DSA. In addition, the microstructures of the surface and fatigue fracture were observed, together with the detection of the surface residual stress. Besides, the residual stresses obtained from the experiment and numerical simulations were compared.

## 2. Materials and Methods

### 2.1. Experimental Material and Samples

A 6061-T6 Al alloy plate of 4 mm thick was used as the base metal for welding. It showed the density, ultimate strength and ultimate yield were *ρ* = 2.672 g/cm^3^, *σ*_b_ = 289.6 MPa and *σ*_0.2_ = 241.3 MPa, separately. ER4043 Al-Si solid wires (Cimic welding consumables Co. Ltd., Shanghai, China) with a diameter *φ* of 3 mm were used. The chemical components of the base metal and the wires are summarized in Table 1.

**Figure 1:**
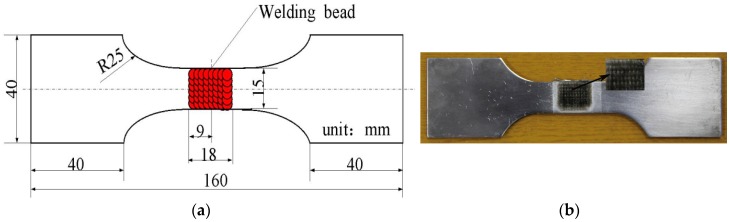
(**a**) Schematic of the specimen treated with LSP assisted by DSA; (**b**) the specimen treated with LSP assisted by DSA.

The base metal was cut into rectangular blocks measuring 220 mm × 40 mm × 4 mm. One end of the block was cut to give a V-typed slope at an angle of 60°, with 1 mm height from lower part of the slope to the bottom of the plate. A SY 250DX Miller argon arc welding machine (Miller Electric Manufacturing Co., Appleton, WI, USA) was applied to weld the metal at a speed of 0.06 m/min under an arc voltage of 14–15 V and a current of 130–140 A. During welding, argon gas was injected at a rate of 15 L/min. Every two rectangular blocks were single-sided welded while being molded on two surfaces. Before welding, a series of auxiliary procedures (descaling, washing using acetone, and clamping) of the welding jig were performed. During welding, plates for arc ignition and stopping were used. After welding, flaw detection was undertaken using X-ray diffraction technology to select the parts without obvious defects and the joints were cut into dog-bone-shaped samples using a wire-cutting machine. Besides, the weld reinforcement was removed, then, No. 240 waterproof abrasive paper (STARCKE GmbH & Co. KG, Melle, Germany) and Nos. 180, 320, 500, 800, and 1000 metallographic abrasive papers (STARCKE GmbH & Co. KG, Melle, Germany) were used to polish the acquired specimens. Finally, the specimens were washed in acetone and then sealed and held for later testing. Figure 1a shows the dog-bone-shaped specimens of the welded joints and the highlighted central region in the figure represents the LSP region.

### 2.2. LSP Assisted by DSA Experiment

**Figure 2:**
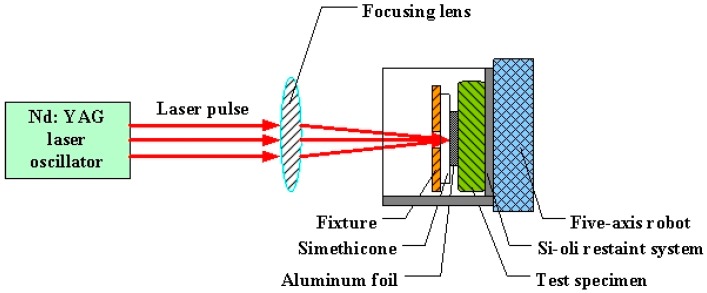
A schematic view of LSP assisted by DSA processing.

Figure 2 shows the LSP assisted by DSA. The Nd:YAG laser used was produced by the GAIA Company in Paris, France and Table 2 lists its relevant parameters. Flexible constraint of laser shock waves was realized by combining PMX-200 simethicone with the self-developed Si-oil restraint system. Al-foil at a thickness of 120 μm was used as the absorbed layer. The temperature control system adopted was a YS-200S system developed by YOTEC Instrument Co., Ltd., Hsin Chu, Taiwan. The laser energy, the diameter of spots, and the overlapping rate were set as 6 J, 3 mm, and 50%, respectively, while the temperature on the surface of the specimen was controlled at 130 °C. The LSP was vertically conducted on both sides of the specimen while it ran horizontally for 11 rows. The corresponding single-surface active zone measured 18 mm × 15 mm. The welded joints treated after LSP assisted by DSA are shown in Figure 1b. In the contrast test of LSP at room temperature, the Si-oil restraint system, and the temperature control system were removed, while the running water was applied as the constrained layer. Other parameters were the same as those in the LSP assisted by DSA. Dual sided LSP were conducted either once or twice. Three welded joints specimens were treated by LSP assisted by DSA, single LSP and double LSP, respectively. 

### 2.3. Measurements and Microstructural Observations

The welded joints before and after LSP assisted by DSA were cut into rectangular blocks to allow the observation of their microstructures. Then, they were continuously polished and eroded using 5% hydrofluoric acid for 1 min. After cleaning, their surface morphology was observed using the XL30 environmental scanning electron microscope (ESEM) (FEI, Hillsboro, OR, USA) and the energy spectrometer, accompanying with the analysis of the chemical components of the precipitates.

The surface residual stress of the welded joints was detected using X-ray diffraction with the sin^2^*ϕ* method [24]. The detector used was the IXRD Combo tester for residual stress produced by the Proto Tool Company in London, ON, Canada, which satisfies the requirements of Europe Standard EN15305-2008 [25] and American Standard ASTM E915-10 [26]. Radiation was performed using Cr-Kα energy on the 311 surface (hkl plane) at a Bragg angle of 139°. Each test point was measured three times with the average value being recorded as the residual stress at this point.

Fatigue performance was tested on an EHF-EG250-40L fatigue tester (Shimadzu Manufacture, Kyoto, Japan), loaded axially with tensile sine-wave loads. The stress ratio R was 0.1, while *σ*_max_ was 100 MPa and the loading frequency was 10 Hz. The temperature rise in the specimen in the fatigue test did not exceed 2 °C. After this detection was finished, the S-4800 cold field scanning electron microscope (Hitachi Chemical Co., Ltd., Tokyo, Japan) was applied to observe the fatigue fracture surface. Three welded joint specimens were selected from three groups, respectively—untreated, and treated by LSP assisted by DSA, and single LSP and double LSP to test their tensile-tensile fatigue life and the average of the values obtained was recorded.

## 3. Results and Discussion

### 3.1. Microstructural Characteristics

**Figure 3:**
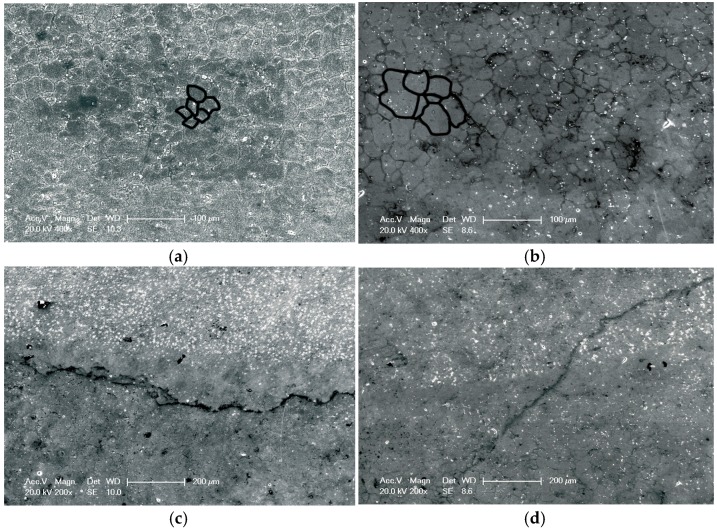
Typical observation in the top surfaces of the specimens with LSP assisted by DSA and the untreated specimens: (**a**) HAZ of the specimen with LSP assisted by DSA; (**b**) HAZ of the untreated specimen; (**c**) TMAZ-HAZ of the specimen with LSP assisted by DSA; (**d**) TMAZ-HAZ of the untreated specimen.

After the weld beads and HAZs of the AA6061-T6 welded joints were treated by LSP assisted by DSA, their surface microstructures were seen to have changed (Figure 3). Figure 3a shows the microstructure of the HAZ of the welded joint after the treatment. As can be seen, the grains were uniform, fine, and about 20 μm in diameter. Figure 3b illustrates the microstructure of the HAZ of the weld joint without LSP treatment, from which it can be found that the grains were coarse and poorly graded. Figure 3c shows the surface microstructure near the weld bead of the welded joint after LSP assisted by DSA; accordingly, a large number of uniformly distributed precipitated phases were observed in the upper weld bead zone, while the lower HAZ was seen to have double uniformly distributed grain sizes. In addition, Figure 3d indicates the surface microstructure near the weld bead of the welded joint without LSP treatment. The left upper weld bead zone showed few precipitated phases with an uneven distribution, while the right lower HAZ was found to have grains of significantly dissimilar sizes.

**Figure 4:**
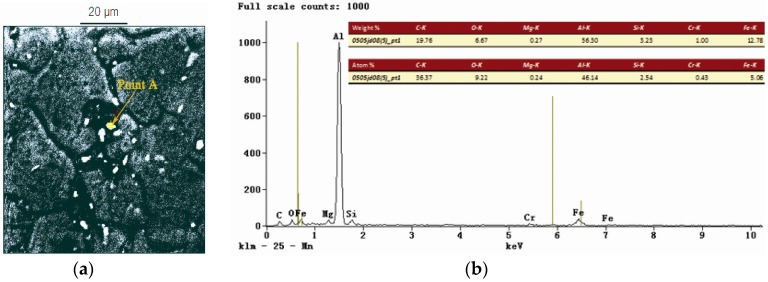
(**a**) EDS map of the HAZ of the specimen treated with LSP assisted by DSA; (**b**) corresponding point EDS analysis marked by arrow A in Figure 4a.

The chemical components of the precipitates from the welded joints were analyzed by using an energy dispersive spectroscope (EDS) and a scanning electron microscope (SEM). The EDS analysis showed that the precipitated phases from the HAZs of the joints after the treatment of LSP assisted by DSA contained magnesium (Mg) and silicon (Si), as presented in Figure 4. That is, the 6061-T6 Al alloy was an Al-Mg-Si alloy. If Mg_2_Si was precipitated in the manner of dispersed phases, the crystal lattices of the *α*(Al) were thus supposed to be distorted. As a consequence, the mechanical performance of the alloy was expected to be significantly improved [27].

### 3.2. Tensile Fatigue Performance

**Figure 5:**
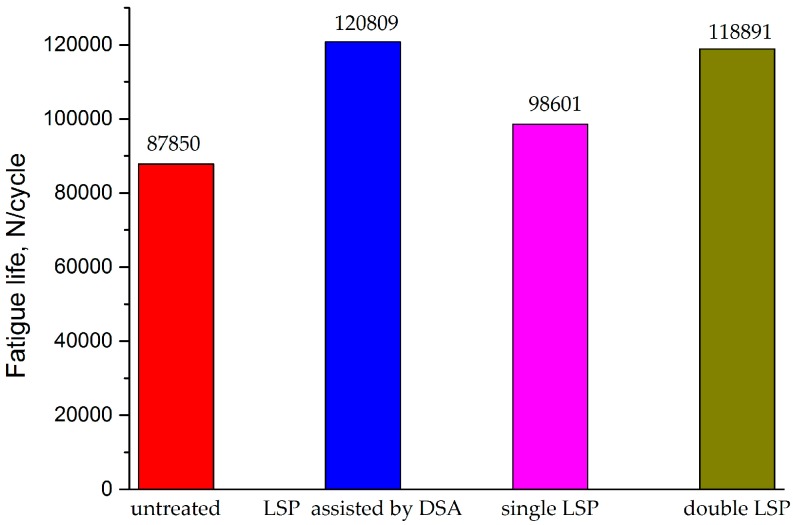
Fatigue life of specimens subjected to different LSP processes.

Figure 5 shows the histogram of the high-cyclic load fatigue life of the AA6061-T6 welded joints after different LSP processes. As can be seen, the specimen which was not treated with LSP showed the shortest fatigue life (87,850 cycles), while the longest fatigue life was found in the specimen treated using LSP assisted by DSA, reaching 120,809 cycles, some 1.4 times that of the specimen not treated with LSP. In addition, the specimen treated once with LSP at room temperature presented a longer fatigue life (98,601 cycles) than that of the specimen not treated with LSP. The fatigue life (118,891 cycles) of the specimen treated twice with LSP at room temperature was further improved compared with that treated once, while it was slightly shorter than that of the specimen treated using LSP assisted by DSA. This is because the high-density dislocation induced by LSP assisted by DSA and the release rate of the residual compressive stress were decreased. Owing to this combined effect, the fatigue performance of the Al alloy welded joints was significantly enhanced, thus exhibiting a far superior strengthening effect to those obtained in single and double LSP at room temperature.

**Figure 6:**
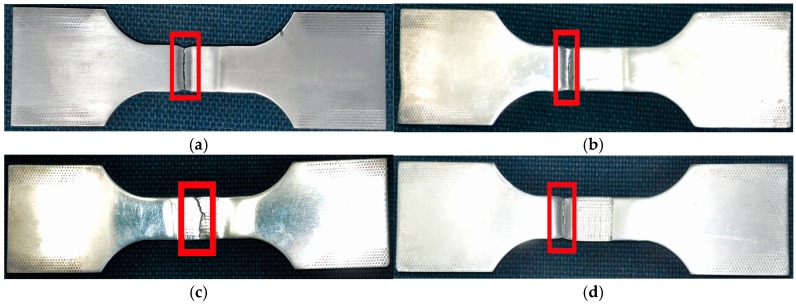
High-cycle fatigue fractures of the specimens subjected to different LSP processes: (**a**) untreated; (**b**) LSP assisted by DSA; (**c**) single LSP; (**d**) double LSP.

Fatigue fractures in the welded joint not treated by LSP were found in the HAZ stretching to straight lines, as indicated in Figure 6a, while fatigue fractures in the joint treated using LSP assisted by DSA were observed in the substrate region outside the strengthened zone in a similar manner (see Figure 6b). Results indicated that, under the effect of LSP assisted by DSA, the fatigue performance of the specimen in the strengthened zone was substantially improved and exceeded that of the base material of the same size. In addition, it was found that the fatigue fractures of the specimen treated once by LSP at room temperature were jagged lines in the central weld bead, as illustrated in Figure 6c. This is because ER4043 wires with good crack resistance were used in the welding process. As they do not contain alloying elements in fine-grain form, the grains in the central weld bead were coarse and the structure was loose. For this reason, pores were readily formed. After the welded joint was treated by LSP, the fatigue strength of both the weld bead and the HAZ was significantly enhanced. However, micro-pores and impurities were still found in the weld bead. Although these were not serious welding defects, the effective cross-sectional area of the weld beads was reduced by the pores, and therefore the weld beads were loosened. The non-uniformity of the material in the weld bead zone accelerated the initiation and growth of cracks. That is, after the welded joints were treated by LSP, the fatigue strength of the weld beads was weaker than that of the HAZs owing to the influence of these less serious defects. Consequently, fractures were generated in the weld bead zone. The specimen treated twice with LSP at room temperature was found to show linear-type fatigue fractures in the substrate region outside the strengthened region, as illustrated in Figure 6d. This indicated that, after double LSP treatment at room temperature, the fatigue performance of the specimen in the strengthened region exceeded that of the base material of the same size. In addition, it was superior to that of the specimen treated once with LSP at room temperature.

**Figure 7:**
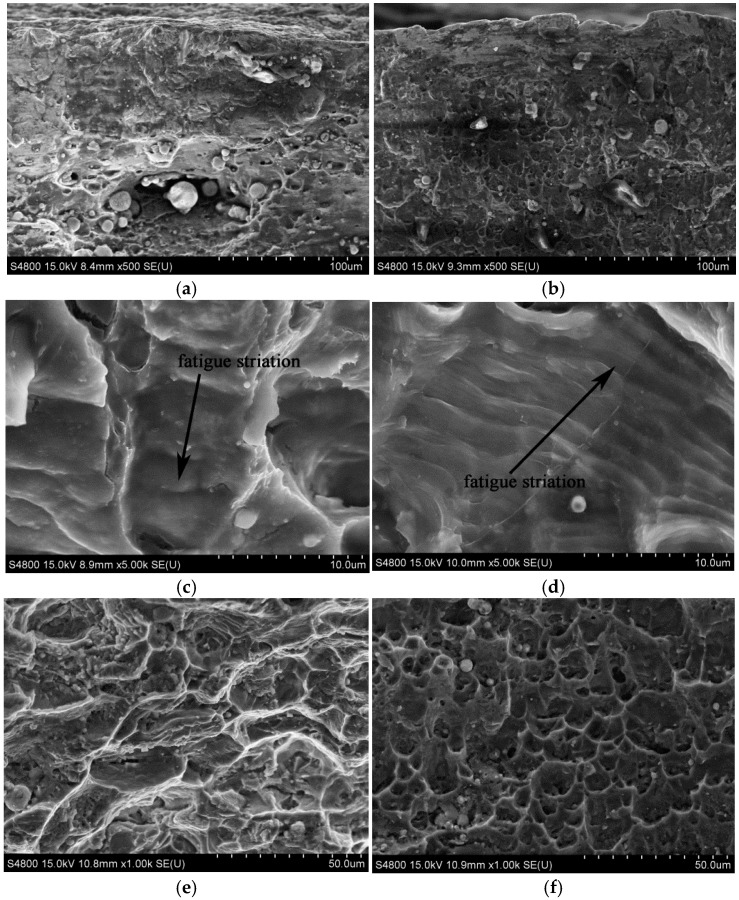
Fatigue fractographs of the specimens with LSP assisted by DSA and untreated: (**a**) crack initiation of the untreated specimen; (**b**) crack initiation of the specimen with LSP assisted by DSA; (**c**) crack growth region of the untreated specimen; (**d**) crack growth region of the specimen with LSP assisted by DSA; (**e**) ultimate fracture region of the untreated specimen; (**f**) ultimate fracture region of the specimen treated with LSP assisted by DSA.

Figure 7 indicates the microstructures of the fatigue fractures of the welded joints before and after LSP treatment assisted by DSA. A large number of crack sources can be found in the fatigue fractures on the surface of the welded joints in untreated specimens, as shown in Figure 7a. After treatment, no crack sources were found on the fracture surfaces and there were few fatigue crack sources on the secondary surfaces (Figure 7b). This is because compact strengthened layers, together with residual compressive stress, were formed on the surface of the specimens in LSP assisted by DSA. Due to this, fatigue crack sources were generated in a downwards direction in the welded defects on the secondary surface instead of from the surface layers. Therefore, fatigue cracks were initiated, thus improving the fatigue life of the specimens. Obvious fatigue strips were found in the crack extension region on the fracture surface of untreated specimens, with their spacing being over 2 μm (Figure 7c). In addition, the crack extension region on the fracture surface of the specimen treated using LSP assisted by DSA was also observed with fine fatigue strips at a separation of 1 μm (Figure 7d). According to the small distance between fatigue strips, it can be inferred that the fatigue cracks on the welded joints extended at a slow rate after the joints were treated using LSP assisted by DSA. Based on Figure 7e, the dimples in the transient zone of fatigue fracture cracks were relatively sparse, while those dimples in the specimen after LSP assisted by DSA (Figure 7f) were deeper and larger, and were distributed densely and uniformly. The larger and deeper the dimples under the same fracture conditions, the better the plasticity of the material. This indicated that LSP improved the plasticity of the transient zone of the specimens. This improvement meant that the generation and extension of cracks slowed down, thus postponing the occurrence time of fatigue fracture. This is conducive to increasing the fatigue performance.

### 3.3. Surface Residual Stress

**Figure 8:**
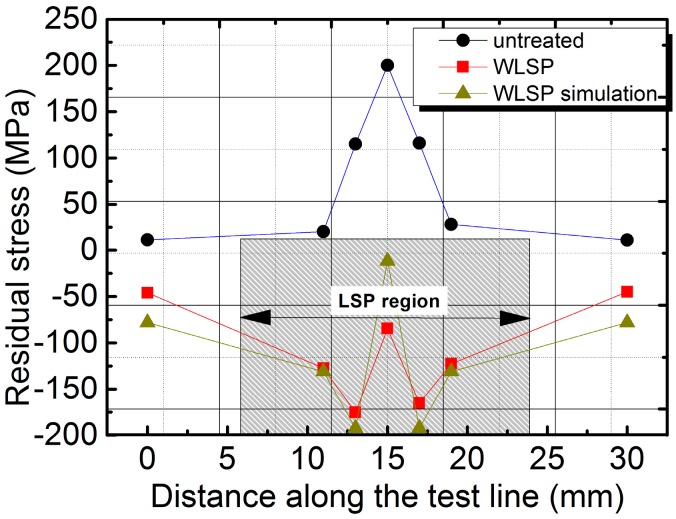
Surface residual stress distributions of specimens.

Three welded joint specimens, untreated and treated using LSP assisted by DSA, were chosen to examine the residual stress regimes therein. The average of the values measured was taken as representative of the residual stress. The distributions of the surface residual stress of the welded joints before and after LSP treatment assisted by DSA are illustrated in Figure 8. Based on this figure, residual compressive stress was present in the whole LSP region after treatment, and the highest stress was found near the weld bead on both sides of the welds, reaching −175.5 MPa. Similarly, the weld beads and the HAZs of the welded but untreated joints were also under a residual compressive stress and the maximum value (200 MPa) was seen in the weld bead. The residual compressive stress gradually fell from the weld bead to both sides of the HAZs.

The detected results of the surface residual stress showed that LSP assisted by DSA can eliminate the surface residual tensile stress on the welded joints and generate a certain residual compressive stress. This was conducive to improving the fatigue life of joints. Meanwhile, both the front and the back faces of the welded joints were found to be under residual compressive stress after double LSP treatment. Therefore, tensile stress was required in the joints to realize the equilibrium of the whole mechanical system [12], while the existence of internal tensile stress was supposed to facilitate the transfer of the crack initiation regions from the surface to the inner side. In this way, the surface performance was improved and thereby the fatigue life of the welded joints was increased.

## 3.4. Numerical Analysis of Residual Stress

Numerical simulation in the TIG welding and LSP assisted by DSA processes was performed using Abaqus software (Dassault Simulia Company, Providence, RI, USA). The user material subroutine (UMAT) was loaded to simulate a moving Gaussian heat source using element death [28], thus obtaining the temperature and residual stress fields after welding. Then, surface heat loads were set in the thermomechanical coupling analyzer and the temperature was defined based on the amplitude of the heat signal. UMAT was programmed in Fortran. The parameters of the moving Gaussian heat source were set as follows: an arc heating rate of 0.85 and an arc voltage and current were selected and supposed to be constant at 15 V and 140 A, respectively, throughout the welding process. Moreover, the width and depth of the heat sources were 6 mm and 4 mm, respectively. The element death approach firstly deactivated some elements in the models. Then, the material properties, such as the density and heat capacity of these deactivated elements, were automatically set to zero, as were the strains therein. Before welding, the dead elements were reactivated to undergo the welding simulation. The activated states of the elements can be defined using Abaqus software.

**Figure 9:**
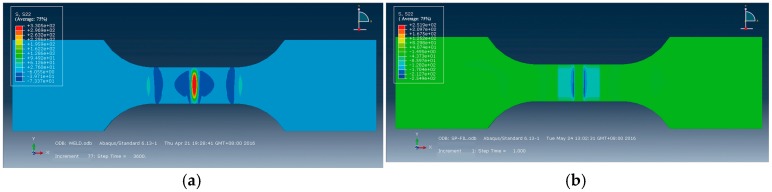
Simulation of surface residual stresses distribution: (**a**) untreated; (**b**) LSP at 130 °C.

Next, pressure loads were applied according to the Fabbro model so as to simulate LSP [29,30]. The simulated residual stress in untreated welded joints is illustrated in Figure 9a. As can be seen, residual tensile stresses were found in the weld beads and the HAZs, and the maximum tensile stress (300 MPa) was found in the center of the cracks. It quickly declined from the weld beads to the HAZs on both sides of the weld beads and tended toward zero on the edge of the HAZs, that is, at a position 6 mm from the weld center. Figure 9b displays the residual stress fields acquired at the working temperature of 130 °C and it was found that residual compressive stress was discovered in the weld beads and the HAZs (LSP region), and the maximal compressive stress was in the weld bond, reaching −192 MPa. According to the comparison of the numerical simulation with the experimental results, the same trend in residual stress was discovered, as shown in Figure 8; however, the tested residual stresses were generally smaller than simulated results due to following reasons:

(1) A minor amount of residual stress might be released as the residual stresses in the specimens, after being treated using LSP assisted by DSA, were not tested sufficiently quickly.
(2) Explicit finite element analysis used in the simulation of the specimens treated using LSP assisted by DSA can generate cumulative errors and the preset parameters, including stepload, might have deviated slightly from the relevant experimental values.
(3) The test results may have been subject to a certain amount of random error.

## 4. Conclusions

This research studied the effect of LSP assisted by DSA on the tensile fatigue life of AA6061-T6 joints welded using TIG from the observation of surface microstructures, the analysis of residual stress, the detection of fatigue life, and the analysis of fatigue fracture. Under the test conditions used, the 6061-T6 Al alloy joints (TIG-welded) were treated using LSP assisted by DSA. They showed an enhanced fatigue life, which was 1.4 times that of unstrengthened joints. The strengthening effect of LSP assisted by DSA was superior to that obtained in single and double LSP treatments at room temperature. Compact strengthened layers were formed on the surface of the joints in the LSP assisted by DSA. As a result, fatigue crack sources moved downwards, and the crack growth rate decreased. The distance between fatigue strips was less than half that in the unstrengthened joints. LSP assisted by DSA eliminated the residual tensile stress on the surface of the joints and the generated residual compressive stress was, at its maximum, −175.5 MPa. Therefore, LSP assisted by DSA is bound to become a promising post-processing method for Al alloy weldments in various businesses.

## Figures and Tables

**Table 1:** Chemical components of the 6061-T6 alloy and the ER4043 welding wires.

Material	Si	Fe	Cu	Mn	Mg	Cr	Zn	Ti	Al
6061-T6	0.61	0.53	0.29	0.11	0.98	0.26	0.19	0.10	Bal
ER4043	4.5~6.0	≤0.8	≤0.3	≤0.05	≤0.05	–	≤0.1	≤0.2	Bal

**Table 2:** Parameters of the Nd:YAG-GAIA laser.

Parameters	Value
Operation material	Nd:YAG
Wavelength/nm	<12
Frequency/Hz	1~5
Power distribution	Flat
Pulse width/ns	<10
Pulse power/J	<12
Focus size, *Φ*/mm	3~8
Spot shape	Circle
